# Educational Participatory Design in the Crossroads of Histories and Practices – Aiming for Digital Transformation in Language Pedagogy

**DOI:** 10.1007/s10606-023-09473-8

**Published:** 2023-06-08

**Authors:** Marianne Kinnula, Netta Iivari, Leena Kuure, Tonja Molin-Juustila

**Affiliations:** University of Oulu, Oulu, Finland

**Keywords:** Digital transformation, Educational participatory design, Language teacher education, Transdisciplinary design education, Transdisciplinary research

## Abstract

Some level of digital technology design skills and competencies is important in any profession but in their education and work life this is often ignored. We explore the potential of Educational Participatory Design (EPD) in transforming work practices within diverse disciplines. This is done through a transdisciplinary case where EPD was used as an approach for transforming language teacher education seen to respond too slowly to technological advancements in society and work life. Based on our findings, we propose EPD as a useful approach for building the design agency of future professionals with various disciplinary and professional backgrounds. In the context of real-life work practice with students as future workers, EPD invites them to act as ‘designers’ envisioning novel practices and technologies for their own work, engaging their ‘users’ in the PD processes. EPD as a novel methodological approach integrates design with work practice learning and education and therefore, we suggest, belongs to the core expertise of CSCW research and design interested in the digital transformation of work practices.

## Introduction

The field of computer-supported cooperative work (CSCW) has since the 1980s explored digital technology for the workplace, and with the fast-paced digitalization of everyday life this focus is even more relevant (see e.g., Pakusch et al., 2021; Sergeeva, 2022). It also calls for increasingly equipping professionals in different fields with skills and capabilities to appropriate, design, and modify digital technologies to suit their needs and purposes.

In the current study, based on a transdisciplinary^1^ case, we explore the potential of Educational Participatory Design (EPD) in transforming work practices of future professionals. This is done through a case of language teacher education in a Finnish university. Future language teachers need to become better prepared to respond to technological advancements in society, and even become change agents in this respect in the field of language pedagogy. The rationale for the case under examination arose from research suggesting that changing pedagogical thinking in relation to current technology-related pedagogical practices seems to be challenging for teachers and pre-service teachers (e.g., Kuure et al., 2016; Fransson et al., 2019; Halkola and Kuure, 2021). There is an ample literature base in educational technology reaching back to the 1990s and an equally long history of societal investment to harness schools with digital technology. Nevertheless, its full potential has not been reached in education, which has triggered a new wave of research and strategic efforts to improve the situation to move from print-oriented practices towards new pedagogies drawing on the affordances of our technology-rich environment (NCCBE, 2016; Pöntinen et al., 2017). The challenges brought by the COVID-19 pandemic underscore the relevance of this topic even more. Teacher education has an important role in enabling university students – change agents of future language pedagogies in schools – to understand how digital technology may change not only the nature of learning and teaching events but also the professional tradition of language education. This transformation must be seen as a complex phenomenon connected with a range of aspects that reach far beyond the school. Historically evolving beliefs, understandings and theories concerning language and language learning have an impact on how our thinking about education, curricula, and the design of pedagogical events proceeds. There are also various traditions of leadership, collaboration, as well as societal policies that may hinder or facilitate digitalization in pedagogical practice.

We adopted a transdisciplinary approach, proposed for handling complex issues (Nicolescu, 2012; Hyun, 2012), to target the complexity of this work practice transformation by combining the Participatory Design (PD) approach (e.g., Greenbaum and Kyng, 1991; Schuler and Namioka, 1993) with language teacher education. Originally, our aim was to develop an educational approach where the language students would become able to design their technology-mediated future work practices (in the role of a ‘designer’) and put into practice a mutual learning and design project for schoolchildren (as ‘users’), drawing on ideas from PD. Our ideal behind the university course organization was that the users (i.e., schoolchildren), in turn, would become active agents, capable of both imagining and participating the design of future technology in any socio-cultural context of their everyday life. Oftentimes, people need to interact with novel artifacts to understand their mediator role and to be able to vision their potential for changing practices, hypothetical new practices acting as important mediators in design (Kuutti, 2013; Tuikka and Kuutti, 2001; Molin-Juustila et al., 2014). We were hoping that mutual reciprocal learning between student teachers and schoolchildren, where ‘designers’ support ‘users’ in taking meaningfully part in the design process, would have provided a path towards that direction.

However, we realized our approach was highly related to the digital transformation of the work practices of language learning and teaching. While conducting projects in a real-life learning and teaching context, the university students had to consider both the technical and non-technical issues of their future work practices experienced in situ. Thus, we started to consider our approach more like an *in-situ* approach for mutual reciprocal learning related to the digital transformation of work carried out as PD in educational (work practice) context, i.e., educational participatory design (EPD).

EPD as an approach, drawing on the tradition of Scandinavian participatory design (e.g., Greenbaum and Kyng, 1991; Schuler and Namioka, 1993), aims at building design agency of future professionals with various disciplinary and professional backgrounds. The purpose is to build their capabilities to transform their future work practices. EPD invites the professionals of the future to act as ‘designers’ envisioning novel practices and technologies and engaging ‘users’ in the PD processes. EPD involves a conscious procedure of learning and viewing the working process on a meta level and involving participants in hands-on experience, reflection and joint sense making. The emphasis is on collaboration between different stakeholders and equalizing power relations, as well as on situation-based actions, mutual learning, and bringing forth alternative visions about technology (see e.g., Greenbaum and Loi, 2012). This includes our strong aim to give the professionals a voice in designing their future work practices. We envisioned EPD to provide a useful mediational means (Wertsch, 1998) for triggering reflection and perspective taking among university students in relation to possible change in their conceptions and practices of language education in their future work.

Our interest at the same time has been to investigate how we, as transdisciplinary researchers, have been able to create this type of a new approach, acknowledging that many contextual factors are intermingled with its implementation. Hence, our research questions are the following: *What kind of resource does educational participatory design provide for transdisciplinary education? What kind of contextual factors are shaping the use of educational participatory design as a resource in transdisciplinary education?* Our theoretical-methodological research approach is nexus analysis with a qualitative and ethnographic baseline. Nexus analysis is considered suitable as it aims at deeper understandings of complex topics during longer timescales (Scollon and Scollon, 2004).

Next, we introduce literature on transdisciplinary research and education in general and in CSCW, as we see the transdisciplinary perspective as central for EPD when CSCW/PD researchers collaborate with professionals from other fields, as well as literature on participatory design as a resource for transdisciplinary education. Then, we present the case involved in this study together with the methodologies we used. After that, we present the findings of the study, and finally discuss their implications for research, practice, and education.

## Related research

### Transdisciplinarity in research and education

Currently, universities are guided towards transdisciplinarity as it has been seen to provide solutions for today's increasingly complex problems also in educational practice (Russell et al., 2008). Indeed, combining views from different fields of research is useful, even vital, as traditional discipline-focused strategies do not necessarily offer sufficient means for understanding and solving complex, multifaceted problems (McClam and Flores-Scott, 2012). In education, transdisciplinarity is considered essential due to the rapidly changing world (Nicolescu, 2012) as it offers us efficient tools in handling complex issues (Hyun, 2012). Therefore, it is an important goal in renewing pedagogical approaches and research (McClam and Flores-Scott, 2012). CSCW research is multi- or interdisciplinary in itself (Crabtree et al., 2005; Bjørn and Boulus-Rødje, 2015). It also offers extensive expertise in studying and supporting work practices of experts representing different disciplines and professions (e.g., Boujut and Blanco, 2003; Lawrence, 2006; Lee, 2007; Schmidt et al., 2009). However, transdisciplinary research, design, or education have received scant attention in the CSCW research tradition. Deepening the understanding of what transdisciplinarity means and how it can be achieved is needed for taking advantage of its potential effectively and genuinely.

Transdisciplinarity in research can be seen in problem focus, methodology, and collaboration (Wickson et al., 2006). As regards problem focus, there is an intention to understand and solve multidimensional and complex problems in transdisciplinary research. Regarding methodology, multidisciplinary research relies typically on discipline-based approaches, each discipline bringing in its own expertise to solve the problem from its own viewpoint (Horlick-Jones and Sime, 2004; Rowland, 2006), using their own theories, tools, and methods (Rogers et al., 2005). In interdisciplinary research, theoretical positions and underlying assumptions need to be negotiated (McClam and Flores-Scott, 2012; Rowland, 2006) and a shared methodological approach needs to be agreed on (Wickson et al., 2006) when tackling with problems that cannot be divided between disciplines (Rowland, 2006). In transdisciplinary research, disciplinary boundaries need to be dissolved to construct methodologies tailored for solving the problem, with an emphasis on the integration of disciplinary epistemologies (Gibbons et al., 1994; Horlick-Jones and Sime, 2004). In transdisciplinary research, collaboration is not only a matter of researchers, as in interdisciplinary research. Rather, it is a matter of intentionally involving also people affected by the research in the problem formulation and in defining the criteria, objectives, and resources that are needed for analyzing and resolving the problem (Klein, 2004). Therefore, knowledge domains from different fields are merged in a dialogue in transdisciplinary work where the differing conceptions, languages, and logics are jointly examined (Horlick-Jones and Sime, 2004; Klein, 2004; Lawrence, 2006; McClam and Flores-Scott, 2012). However, some overlap in the participants’ knowledge bases is needed for finding a shared problem focus. Eventually, the participants need to develop mental models they share as well as a common knowledge base (Godemann, 2008).

When now looking back to the problem focus, even though multi- and interdisciplinary research can have a common goal and contribute to solving a shared problem, it is necessary to cross disciplinary borders in order to create new knowledge (Rowland, 2006). This is important not only for problem identification and problem setting but also for sharing and integrating disciplinary epistemologies through long-term collaboration. Teachers and researchers striving for transdisciplinarity often meet with challenges as the practice requires flexibility (Russell et al., 2008). Universities and disciplines still tend to act in their own domains even when the explicit goal is crossing disciplinary boundaries (Pharo et al., 2012).

Certain factors seem to contribute to the success of the transdisciplinary approach. The attitude of the participants is central. They need to be willing to openly question their existing (disciplinary) modes of thinking and be ready to learn from other disciplines (McClam and Flores-Scott, 2012; Wall and Shankar, 2008). They also need to be humble and brave when encountering new views and approaches that threaten to break the familiar structures of thought and action (Wall and Shankar, 2008). Debates between the participants are crucial in opening the thoughts and providing space for growth (Wall and Shankar, 2008). Physical, administrative, financial and social resources are important in supporting the work (Wall and Shankar, 2008). As Godemann (2008, p. 637) suggests, success in a knowledge integration process arises from a) exchanging information, b) achieving understanding, c) creating a common knowledge base, d) achieving awareness of the frame of reference, and e) developing group mental models. Thus, turning trajectories towards new paths requires openness, critical reflection, dialogue, and collaboration.

In the context of teacher education, transdisciplinarity has been a goal for some time. In transdisciplinary education, co-teaching and co-mentoring have been found to be fruitful in integrating disciplinary perspectives as in developing inclusive practices among teachers in early education (Silverman et al., 2010). Many teacher education programs are fragmentary and incoherent, which hinders reaching the aims of educating student teachers to address real-life problems through transdisciplinary instruction (Mueller Worster and Rohde, 2020, p. 208). Research on arts, science and computing teachers collaboratively creating a learning environment shows how they were able to combine their disciplinary interests, values, epistemic criteria, and practices efficiently in their design work (Finch et al., 2021). In that study, disciplinary boundaries and school structural challenges were surpassed and synergies and tensions in disciplinary integration were critically explored.

In applied language studies, where research on language pedagogies and language teacher education can be situated, transdisciplinarity has been dealt with quite extensively, except for the field of computer assisted language learning and teaching (Hubbard and Colpaert, 2018). Considering ongoing pedagogical change related to but not necessarily caused by digitalization, teachers face challenges in contemplating the renewal of the curriculum and learning culture. As the digital resources available are replaced with others, teachers may experience their own pedagogical approaches conflicting with the new environments although strong familiarization with digital technologies may facilitate their ability to adapt to new situations (Fransson et al. 2019, p. 114). Depending on the nature of the communities they work in, and their personal histories as teaching professionals, teachers may also be in different positions to benefit from digitalization and promote it (Fransson et al., 2019).

Multi-stakeholder and multi-discipline collaboration and construction of how we see the world are in the heart of computer-supported cooperative work (CSCW), and the field has been influenced and transformed by a range of disciplines through its lifespan (Schmidt and Bannon, 2013), resulting in a heterogenous and interdisciplinary field of research (Bjørn and Boulus-Rødje, 2015). Hence, it is no surprise that CSCW research is interested in multi-, inter- and cross-disciplinary research and practice (e.g., Cummings and Kiesler, 2003; Cummings and Kiesler, 2008; Nomura et al., 2008; Miller et al., 2014; Robb et al., 2016; Lee et al., 2006; Kraut et al., 1987; Jackson et al., 2011; Lawrence, 2006; Lewkowicz et al., 2008; Kane and Luz, 2009; Ylipulli and Luusua, 2019). There is also an interest in CSCW to explore how CSCW and education can together benefit both disciplines (Wardrip et al. 2013).

Studies explicitly addressing multi-, inter- and transdisciplinary research and education are still rare in CSCW. Sanner and Øvrelid (2020), Kee and Browning (2010), and Pennington (2011) examine transdisciplinary work among researchers or other professionals, but not transdisciplinary education. Some attempts to that direction exist in the context of architectural education (e.g., Iacucci and Wagner, 2003; Molin-Juustila et al., 2014). Students’ experimental and explorative work with prototype technologies has been discussed in these studies as ‘an enculturation in’ professional architectural design practices. Students have also been positioned as ‘representative users’ or ‘potential future users’ while experimenting ‘in the wild’ with architectural planning tools ‘supposedly supporting emerging new work practices.’ In these studies, design agency among the students has not been in focus, however. Human–Computer Interaction (HCI) research, on the other hand, has introduced transdisciplinary design as the fourth design paradigm, alongside the technical, cognitive, and ethnographic paradigms, to approach interaction design education, scholarship, and practice (Blevis et al., 2015; Moore and Lottridge, 2010; Blevis and Stolterman, 2009). We see PD as providing significant opportunities for developing transdisciplinary research, design, and education further.

There are arguments in CSCW suggesting that future research is needed as previous studies have shown that multi-, inter- and cross-disciplinary work is challenging (Boujut and Blanco, 2003; Lawrence, 2006; Lee, 2007; Schmidt et al., 2009). Communicating, collaborating, and arriving at shared understandings may be difficult (Boujut and Blanco, 2003; Lawrence, 2006; Lee, 2007). As Lee (2007, p. 335) puts it: ‘Collaborative work can be highly contested and practices and artifacts are not always well understood. Alignments can be partial, shared understanding between groups can be spotty, and these breaks in alignment extend to understanding and use of representational and coordinative artifacts.’

### Participatory design stance as a resource for transdisciplinary education

PD has gained attention within numerous disciplines as a concept and fruitful approach to design. Its origins lie in the Scandinavian tradition in systems design that held workplace democracy as its goal and considered union involvement vital in developing computer systems in the workplace (e.g., Bjerknes and Bratteteig, 1995; Greenbaum and Kyng, 1991; Schuler and Namioka, 1993). In the course of time, political emphasis has somewhat decreased and fostering the participation of the skilled user in the design process has moved to the foreground. Along this trajectory, the design process has been conceptualized as cooperative work, which implies that designers and users together envision and design users’ future work practices and digital solutions, valuing each other’s expertise and skills. Greenbaum and Loi (2012), and Luck (2018) list eight guiding principles for participatory design (Table 1) and argue that they still stand. The design process requires ‘mutual reciprocal learning’ and ‘design by doing’ where both designers and users learn. Especially users need to be carefully supported to meaningfully take part in the design process (e.g., Bjerknes and Bratteteig, 1995; Greenbaum and Kyng, 1991; Schuler and Namioka, 1993).

**Table 1:** Participatory design guiding principles.

Principle	Explanation
Equalizing power relations	Those who are invisible or in a weaker position in the power structures should also be given a voice (Greenbaum and Loi, 2012). With this aim of everybody having a chance to participate PD has a ‘social agenda’ for design (Luck, 2018)
Situated action	PD ‘is rooted in a concern for located accountabilities (Suchman, 2002), where each project is contextually relevant, meaning that each application is embedded and is designed/crafted to suit local characteristics and circumstances (Simonsen et al., 2014).’ (Luck, 2018) Thus, in PD it is central to work ‘directly with people and their representatives in their workplace or homes or public areas to understand actions and technologies in actual settings, rather than through formal abstractions’. (Greenbaum and Loi, 2012)
Mutual learning	Participants of the PD process are encouraged to understand each other, and this is enhanced ‘by finding common ground and ways of working’ (Greenbaum and Loi, 2012). Participants typically undertake the roles of users and designers, with the aim of the designers striving to understand the users’ situation and the users striving to articulate what their aims are as well as learning the needed technological means (Robertson and Simonsen, 2013)
Tools and techniques that support the actors and the work	The aim of the tools and techniques used within PD is to ‘support collective ‘reflection-in-action’ to enable participants to participate in design’ (Luck, 2018) and to help them to express their needs and visions (Greenbaum and Loi, 2012)
Infrastructuring	The concept of infrastructuring in the PD context (Karasti, 2014) emphasizes the configuration of the situation, the need for tools and methods (Robertson and Simonsen 2013, p. 5) and the constant ‘becoming’ state of the infrastructure (Karasti, 2014)
Shaping the future through alternative visions about technology	PD, like any area of design, aims for producing something or changing the future (Luck, 2018). For this purpose, users of information technologies should ‘play a critical role in their design’ (Robertson and Simonsen, 2013) as ‘[i]f we are to define the futures we wish to live, then we need those whose futures they will be to actively participate in their design’ (Robertson and Simonsen, 2013)
Democratic practices	PD can be seen as a set of values where the idea of democracy ‘leads to considerations for proper and legitimate user participation’ (Binder et al., 2015, p. 163). In a researcher, this set of values manifests as ‘a conviction, personal volition and research stance’. (Luck, 2018) In the design process, this shows as ‘putting into play the practices and role models for equality among those who represent others’ (Greenbaum and Loi, 2012)
Genuine participation	Genuine participation as a concept is in the heart of PD, capturing the ‘fundamental adjustment of the users’ role from being merely informants to being legitimate and acknowledged participants in the design process (Robertson and Simonsen 2013, 5).’ (Luck, 2018)

Recently, the political interests in PD have been revitalized: there is an increasing interest in enabling and supporting PD in various everyday life contexts that are seen as complexly intermingled with power and political issues (e.g., Björgvinsson et al., 2010; Le Dantec and DiSalvo, 2013; Simonsen and Robertson, 2013). Overall, PD research and practice have focused on the design process, giving voice to those who will enact the emerging new activities supported with envisioned new digital technologies – with political agendas and emphases of different kinds. That is not straightforward as many challenges for PD have been identified since its early days. These include contextual factors that affect the PD work (Kraft and Bansler, 1994) and the parties' varying motivations and agendas (Krüger et al., 2021).

Literature on the use of PD in the field of education is scarce. Available studies emphasize new technologies, especially web 2.0, as transforming teaching and learning, enabling learners to become more active participants as well as to act as knowledge and content creators (McLoughlin and Lee, 2008; Palaigeorgiou et al., 2011; Wang and Kao, 2013). Learners have been involved in designing new learning environments and technologies (Koehler et al., 2004; Palaigeorgiou et al., 2011; Wang and Kao, 2013). Additionally, learners and teachers have collaborated in different kinds of teaching development endeavors (Cook-Sather, 2014; Duah and Croft, 2011; Seale et al., 2015); learners have created learning materials, encouraged and provided feedback to teachers in the role of activists in order to improve their learning experiences as well as engaged in curriculum renewal in collaboration with the staff. Learners have even been invited to the steering groups of projects to truly empower them, making their voices heard. In the field of learning research, there are different approaches drawing on the idea of PD more or less loosely (DiSalvo et al., 2017). However, the studies drawing on PD mostly focus on the collaborative process producing an outcome, such as a learning environment, rather than developing the mindset of participants, i.e., the full-fledged PD process where teachers start to act as designers of the digital future, envisioning and designing novel technologies and practices, in collaboration with learners (‘users’), (Kuure et al. 2016 as an exception). Some studies point to that direction, such as Polman et al. (2017), examining teachers and researchers working together to develop children's science literacy.

Even if discussions on agency and power have not been in focus in CSCW research, the potential of combining CSCW and PD has been identified (Kuutti, 2013). Design is one of the cornerstones of PD and we consider offering design education for future professionals within various disciplines as pivotal in the digital age. We see this as a promising new area for implementing the original PD philosophy, i.e., to focus on ‘the social agency and institution-shaping behavior’ while enabling future professionals ‘to participate in the shaping of the worlds in which they act’ (Blevis and Stolterman, 2009; Simonsen and Robertson, 2013). As the use of digital technology is expanding, PD in educational context becomes relevant for various disciplinary and professional domains when the professionals struggle with how to make use of the new digital technologies. This is where work practice oriented CSCW brings value (Kuutti, 2013; Bjørn et al., 2016). In addition to design orientation, there is a strong emphasis on mutual learning to understand the emergent realities of different types of technology-mediated work settings as well as on the co-design of the future work practices; i.e., presence and change intertwined (Karasti, 2001). With the EPD approach suggested in this paper, university students potentially learn to regard themselves as ‘designers’ who can shape technology and its trajectories, not only to adopt it as such. In addition, they potentially learn to engage others as ‘users’ in the shaping of digital technology and its trajectories.

## Methodology

This study arises from long-term transdisciplinary research collaboration that joins together perspectives of CSCW, PD, and human–computer interaction (HCI) (‘technology researchers’) and applied language studies (‘language researchers’). Our focus of research has been people’s agency in everyday technology-rich life. Our shared theoretical-methodological approach, nexus analysis, is a multidisciplinary research framework interconnected with practice and activity theories, linguistic anthropology, discourse analytic studies, and interactional sociolinguistics (Scollon and Scollon, 2004; Scollon and de Saint-Georges, 2013). Social action is seen as emerging from a nexus of ‘semiotic cycles of people, discourses, places, and mediational means involved in the social action’, and their trajectories – in micro, meso, or macro level – meeting in this nexus of practices (Scollon and Scollon, 2004).

### Case context

We conducted a master’s level university course for student teachers, who were studying English as their major subject in a Finnish university, to become English language teachers. The course involved designing an English as a Foreign Language (EFL) project for 11–13-year-old schoolchildren at a local school (Table 2).

**Table 2:** Study participants.

Participants	Role
Cross-disciplinary research group	6 researchers with various backgrounds: technology research (3), language studies (3, including the teacher responsible for the course, two assisting)
University students (student teachers)	12 master’s-level students of English attending the course; 6 oriented to becoming language teachers, others otherwise interested in the course topic; from now on called as ‘student teachers’; acting as ‘designers’ of the future of language learning and teaching
School teachers from a local school	3 teachers with their schoolchildren; not involved in designing the university course or the EFL project but monitoring the children’s activities at the school from a distance
Schoolchildren from a local school	59 Finnish-speaking schoolchildren (aged 11–13) studying English as their foreign language in a local school; taking part in the project as ‘users’ of new language learning methods

Although the scenario of technology use in language education is increasingly moving towards environments that afford new working methods and learning paths, technology is still often portrayed as an add-on in the classroom without considering its pedagogical value (see e.g., Blin and Jalkanen, 2014; Pöntinen et al., 2017). The course aimed to lead the student teachers to consider how language education and their own professional practices and profiles would transform with the change of technology. The course had been repeated over several years with the aim to trigger change in the participants’ thinking about the future of language learning and teaching. It had proved difficult for students to distance themselves from habitual language teaching practices and orient to the future (see Tumelius and Kuure, 2021, Tumelius et al., 2022), due to prevailing print-oriented teaching practices and classroom-oriented emphasis in teacher education among others. The EPD approach was seen to be a potentially effective way for advancing the goal.

Various action methods resembling typical PD methodologies had previously been used on the course for triggering imagination and detachment from the traditional classroom environment and ways of learning and for supporting the students’ brainstorming and envisioning on future technologies and practices of language pedagogy. The problem-based approach including concept design and user analysis had also been used in planning language learning projects for schoolchildren. However, these methods had not been used in the systematic context of an EPD approach so far.

### Course planning phase

The general learning objectives for the course were to familiarize the student teachers with the possibilities of new technologies for EFL teaching and giving the student teachers practical possibilities with working in an authentic school environment with schoolchildren. A central objective was to help the student teachers to see the schoolchildren as collaboration partners in the process of planning their own learning. Our research group worked together planning how the EPD approach could be used to renew the course and allow the student teachers to see themselves as ‘designers of language learning with technology’, rather than as traditional language teachers building their teaching primarily around the textbook with familiar types of exercises on linguistic matter. The idea was to invite the student teachers to enact in a participatory ‘designer’ role, envisioning and taking actions towards the future and seeing their pupils as ‘users’. In the spirit of PD, these ‘users’ were supposed to be engaged in active envisioning of the language learning practices in a technology-rich environment. The student teachers were supposed to ideate concepts for the future technologies for language learning both on their own as well as reflecting on them with the schoolchildren. Further, the goal was to learn reciprocally through design by doing (Greenbaum and Kyng, 1991; Schuler and Namioka, 1993). The PD principles and practices implemented in this course were geared towards supporting both designers (i.e., student teachers) and users (i.e., schoolchildren) to meaningfully take part in the design process (e.g., Schuler and Namioka, 1993; Bjerknes and Bratteteig, 1995; Greenbaum and Kyng, 1991). The course involved training for the designers to enable them to carry out the design process that entails inviting their users to designing their own learning process (for guidelines, see e.g., Druin, 2002).

The EPD approach was initiated by one of the researchers conducting a pre-survey among the schoolchildren who were later to take part in the theme week that the student teachers would design. The survey mapped schoolchildren’s interests and viewpoints related to language learning with technology (see Kuure et al., 2016). The idea was also to raise schoolchildren’s interest and prepare them for the coming studies. The schoolteachers were also consulted. In this phase, it was not possible to include the student teachers as their course had not started yet, even though this did not follow the PD spirit.

### Course implementation phase

The university course implementation included a six-week procedure of five steps that all contained workshops and team meetings leading to work online: 1) orientation, 2) developing ideas and getting prepared, 3) theme week at school with schoolchildren, 4) specifying concepts for future language learning, and 5) the review of the concepts and reflection including written project reports. Three researchers of language studies from the research group were involved in practical arrangements, one of them as the teacher responsible for the course. They also took part in the theme week activities at school. The three technology researchers were engaged in supporting the student teachers in their background research. They oversaw the PD introduction and provided their expertise in considering fruitful technology examples for inspiration. They also had a central role in the stage of concept presentations and analysis.

At the beginning of **Step 1**, the student teachers were introduced to the principles of PD in general and schoolchildren as design partners in particular, the approach for the course, and the findings from a pre-survey conducted with the schoolchildren at the participating school. They were also presented with some examples of innovative technologies. Future language learning and technology development were envisioned together in all aspects, not just in relation to formal school, to help them broaden their thinking about language pedagogy. The student teachers also got acquainted with children’s learning, their language learning practices as well as the technologies and social media they were familiar with.

In **Step 2**, the student teachers were further developing ideas for the activities to be applied during the theme week. The schoolchildren were supposed to engage in various activities using English but also engage in imagining as concretely as possible how they might use and learn languages in future.

During the theme week at the school (**Step 3**), the student teachers worked together with the schoolchildren in different workshops, putting into practice the activities planned in the previous steps.

After the theme week, in **Step 4**, the student teachers were expected to reflect on the experiences gained and develop their concepts for future language learning further, to collect a ‘toolbox’ for their future work as language teachers. They presented their new concepts, which were then discussed and reviewed together in the last course session. The whole research group took part in the discussion, which included an analysis of the PD approach.

Finally (**Step 5**), the student teachers wrote their reflections on the issues dealt with during the course (descriptions on the work completed, lessons learnt, and visions for the future).

### Data and analysis

This study emerged naturally from our long-term collaboration on technology use in people’s everyday life and we took the transdisciplinary lens into use only retrospectively. The qualitative data analysis that results in the findings presented in this paper has happened in many cycles over a couple of years in the process of sense-making and meaning making, researchers working both individually as well as collaboratively in several data workshops to analyze the data as well as to reach a shared understanding through extensive discussions.

The diverse data were generated in three contexts, the university course itself, the theme week at school (‘school project’, which was a part of the university course), and research group analysis workshops after the course (Table 3). In the current study, we are specifically interested in the working processes related the course, not the design outcomes of student teachers or schoolchildren.

**Table 3:** Collected data.

Type of data	Data source
University course	
Course planning material and memos	Research group / Planning workshops
Pedagogical materials (session schedules; gradually constructed web workspace, tools for activities, guidelines, agendas and logs)	University teacher / Course sessions and web workspace
Discussions and brainstorming materials concerning the implementation of the project	All participants / Web workspace
Instructions and materials for the school workshops	Student teachers / Web workspace
Presentations of the concepts for future language learning	Student teachers / Course session
Reflective writings	Student teachers / Web workspace
Project reports (reflections incl. lessons learnt)	Student teachers / Web workspace
Observations and fieldnotes of activities	University teacher
Theme week at school	
Video recordings of activities	Schoolchildren, student teachers and university teachers / Workshops (9.5 h)
Audio recordings of activities	Schoolchildren, student teachers and university teachers / Workshops (4.5 h)
Text documents of generated stories	Schoolchildren / Workshop
Video clips of dramatized dialogues	Schoolchildren / Workshop
Sketches of NFC games	Schoolchildren / Workshop
Observations and fieldnotes of activities	Researchers / Workshops and school
Research group work after the course	
Memos	Researchers / Various meetings and workshops, including data analysis workshops
Reflective writings	Researchers

In the first phase of data analysis, the aim was to go through the data and understand what happened during the course. This resulted in writing a paper focusing particularly on the challenges the students faced in the course from their future profession perspective (Kuure et al., 2016).

In the second phase of analysis, for the purposes of the current study, we focused particularly on our transdisciplinary collaboration process and EPD as an interesting result of that. First, we identified from the course planning and implementation documentation the PD elements that were used on this course. Next, we navigated the material produced by the student teachers during the course to shed light on how well the student teachers seemed to apprehend and utilize the PD principles and practices in their work, using the existing PD literature (see Table 1) as a sensitizing lens. In the third phase, we considered how well we altogether managed to carry out the transdisciplinary education process, based on the understanding from the previous phase of analysis and reflecting our findings to the literature on transdisciplinary work discussed in Sect. 2. Here, the outcome of this work was also examined from the perspective of language teacher education: whether the PD approach had increased student teachers’ understanding of their professional future in utilizing digital technology in language teaching. The focus was on how well we were able to integrate the transdisciplinary research, design, and education perspectives into the work in the case, what kinds of successes and challenges were encountered, and what were the possible reasons for the challenges. This sense making process led to characterizations of these aspects in Table 4 in Section 4.1. Due to the identified challenges, we realized a need for more in-depth understanding of the intermingled contextual factors. Hence, we utilized nexus analysis to sensitize us to various social and historical aspects intertwined with any social action as well as linkages reaching beyond situated action (Scollon and Scollon, 2004; Patton, 2014), including transdisciplinary education with EPD as a resource. In this phase, the perspective of trajectories and practices was contemplated in more detail with reference to participants’ histories, relationships and discourses emerging. Finally, we engaged with the extant CSCW discourse on work transformation with digital technologies: what potential EPD and the transdisciplinary approach have in enhancing our understanding of future digitalization in other professions, and how practice based CSCW research (Bjørn et al., 2016; Kuutti, 2013) can be related to this effort.

As our research approach draws not only on diverse types of data but also researchers’ and university teachers’ experiences and observations, advancing through multiple cycles of analysis to achieve a deeper understanding of the phenomenon being studied, all the interpretations cannot be supported by extracts from data. Rather, the credibility of the study arises from a thorough and critical description of situations and our reasoning.

## Findings

Next, we present our findings with the focus on PD as a resource in transdisciplinary education.

### Successes and challenges when trying to follow the PD principles

Table 4 illustrates our reflections on applying EPD on the course and at the theme week at school, related to the PD principles (see Table 1) that emerged as relevant in relation to the university course in our data analysis. Table 4 also shows how the university course had its own aims and challenges, and the student teachers working at the local school in the ‘school project’ had their own ones.

**Table 4:** Successful and problematic aspects in EPD and at the theme week.

on the university course	in the school project
PD Principle: Equalizing power relations	
+ PD supported the course approach of collaborative teamwork emphasizing equal relationships in teamwork - course teacher’s and researchers’ voice should have been stronger in the planning phase to direct the designs towards new practices	+ student teachers redesigned traditional school activities to increase collaboration and agency - student teachers positioned schoolchildren as pupils, not as co-designers - schoolteachers took no active role in planning so their relationship with student teachers remained thin
PD Principle: Situated actions	
+ PD provided a motivating environment for student teachers to envision the future - student teachers’ design was burdened by viewing language learning as a skills-based rather than a creative situated process	+ student teachers adapted their designs when interacting with the schoolchildren - pedagogies arose mainly from familiar school practices, not from design activities - restricted time reduced student teachers’ possibility to fully understand situated circumstances at school
PD Principle: Mutual learning	
+ course approach allowed negotiation for meanings when new perspectives were reached for - technology researchers had limited time to devote to collaboration with student teachers - student teachers did not draw on insights gained from schoolchildren when refining their concepts	+ pre-survey at school provided student teachers with insight into schoolchildren’s world + student teachers treated schoolchildren as active agents engaging them in learning - student teachers had no contact with schoolchildren at school in advance
PD Principle: Tools and techniques that help participants express their needs and visions	
+ student teachers appropriated the project- driven approach easily as their own study method + course aim involved finding new practices (methods and tools) for future language learning - lack of available collaborative applications suitable for children constrained student teachers’ pedagogical designs	+ schoolchildren’s familiarity with the new school building allowing flexible learning with digital technology made it a natural space for the project - technology-domination on classroom walls and in other learning spaces hindered full use of project-based activities: posters needed for group activities were placed on tables, which changed the nature of interaction and activity
PD Principle: Alternative visions about technology	
+ student teachers’ previous understanding of digital media from out-of-school contexts became visible - student teachers were not fully able to create a link between the results of technology ideation and the design of concrete activities at the school	+ difficulty of technology-ideation with children became tangible to student teachers - technology-ideation with schoolchildren only took place in one workshop where a researcher familiar with the tradition was present
PD Principle: Democratic practices	
+ student teachers adopted a student-centered approach with a balanced relationship between participants + course design highlighted democratic distribution of labor and decision making - the working process related to PD was not made sufficiently explicit on the course	+ long-term communication between the university course organizers and the school helped to establish a configuration to fulfil the objectives of all stakeholders - constraints related to time and study pressure reduced student teachers’ full participation in organizing schoolwork
PD Principle: Genuine participation	
+ genuine participation of all stakeholders was a conscious aim on the university course - technology researchers could have highlighted PD ideals more - school participants were not engaged in planning - student teachers did not finalize their concepts drawing on schoolchildren’ ideas	+ PD experience helped student teachers value schoolchildren’s active participation - schoolchildren’ engagement in design was low - all technology researchers were not present at school - moving between locations was difficult for student teachers hindering their ability to work with children

The PD principles were met with in many respects, but there were also challenges. There was a strong emphasis on *equalizing power relations* among the participants. EPD aligned well with the previous course approach and brought in new elements emphasizing the importance of involving users. The student teachers designed school activities for the schoolchildren building on their interests paving way for them to gain agency. However, the schoolteachers' role remained rather marginal, monitoring what was going on than participating actively in the planning phase. Thus, the potential of schoolteachers' mentoring input did not materialize. As the theme week designs did not differ greatly from regular school activities and the schoolchildren were not positioned as users engaging in envisioning the future, the course teacher and the researchers concluded that their support should have been stronger.

*Situated action* became foregrounded in the school project in providing the pedagogical environment and the process of PD as the foundation for work. However, shaping the school project to align with these aims was hindered by situated constraints governing the student teacher's time available. The degree studies involve a range of different courses in different disciplines, in different ways in the case of each student teacher. Thus, the configurations of schedules combined with the complexity of schedules at the school limit the space for action with the schoolchildren and teachers. However, the student teachers appeared to be able to flexibly adjust their ongoing pedagogical situated action when seeing how the schoolchildren responded to their approaches.

*Mutual learning* was also realized in the course being based on balanced relationships and learning by doing together. This was strengthened by an attempt by the student teachers to find out more about the schoolchildren, which, however, remained at the level of one pre-project survey and the information mediated by the course teacher from the school to the student teachers. Time restrictions, again, reduced the possibilities to build on mutual learning more strongly, although on a general level, this aim was, indeed, reached reasonably well.

Regarding *tools and techniques* that help the participants to express their needs and visions, the student teachers adopted the project-driven approach and EPD easily in terms of the working method although the user-focus was weak. Searching for new practices, including the use of new tools and methods, was aimed for but the goal was reached only partially due to the lack of suitable collaborative applications and online environments usable with young children. Because of the city decisions on the allocation of funding the school had certain applications available, but their use was not possible for the project for reasons related to user management (of pupils and users coming from outside the school).

In terms of triggering *alternative visions about technology*, student teachers appeared to be fluent users of social media and technologies for their own purposes, but it was challenging for them to link such expertise with pedagogical goals. The project did bring into foreground the difficulty of technology ideation with children, which would strengthen the student teachers' understandings and practices in the future.

*Democratic practices* were advanced through the aims of the course to involve participants with equal opportunities to participate in the planning and activities. However, practical circumstances related to time and pressing schedules hindered the full participation of the student teachers. The complex configuration of timetables required considerable effort to arrive at a flow of activities that was doable for everyone.

Ensuring *genuine participation* was a conscious aim on the course, as the pursuit for equalizing power relations, mutual learning, and democratic practices also suggest. As explained above, there were various elements in the student teachers’ life situations as well as the institutional circumstances that made it difficult to put EPD into practice in its ideal form, engaging the users on a long-term basis. The researchers and the course teacher could also have put more emphasis on supporting the student teachers in adopting the designer role as well as in engaging the schoolchildren in that role according to the PD aims. An important aspect on the course was, however, that the student teachers had the opportunity of considering ways to engage schoolchildren in genuine participation, which is important for their future professional practices in the field of language pedagogy.

### Educational participatory design in the crossroads of histories and practices

There were diverse issues at play in terms of how the EPD process advanced. The actors had varying agendas and their personal histories affected their practices – what they do and how they do it. The contexts – university and school – have their own practices (many of them for historical reasons), embodied in the participants, limiting, and guiding them toward certain directions.

#### Diverse agendas in the Flux

**Figure 1:**
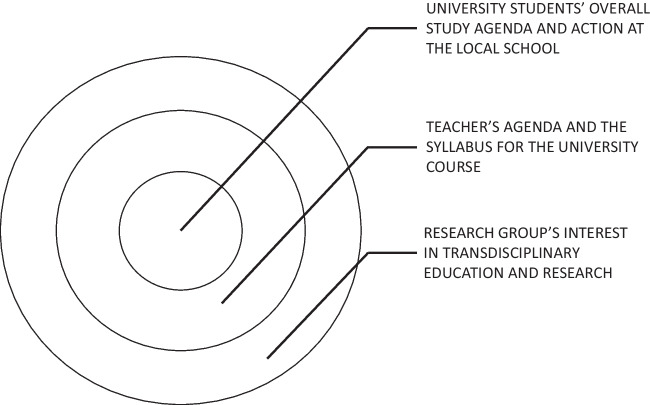
Diverse agendas in flux.

Figure 1 illustrates the complex configuration of agendas (with varying degrees of awareness) that each actor in the project drew on in their participation in the EPD project. On the surface, the setting seems simple – the university students (student teachers) are taking a course that is part of their degree studies, the university teacher is responsible for offering a design-driven course that promotes student teachers’ growth to language teachers in the technology-rich world, and the research group bringing in the EPD perspective to facilitate the PD ideals, which is also the wish of the teacher. As the findings above suggest, the picture is, however, far more complex than expected. Fitting together the school timetable with the timetables of individual student teachers with their personal subject combinations as well as the framework allowed by the university course was not simple. Student teachers also had to calibrate their studies with their overall life and time management and preferences, switching between different positions (e.g., student, designer, teacher, parent, student teacher). The university teacher needed to adhere with the pedagogical course objectives for the university course, while functioning as a hub between the schoolteachers, the research group, and the student teachers with their varying life situations. The research group provided support for the course teacher to push action to follow the EPD process facing the pressures above, but many of the goals of the research group and the university teacher were downplayed due to these obstacles produced by structural factors manifesting in the daily work practices.

More distant agendas echoed in the working at school as well: The school followed the city agenda for modern ‘smart schools’ and it was thus planned for modern pedagogy with its open and transformable spaces and furniture. This high-level agenda affected the working practices at school: the modern design and technology at the school offered a flexible space making it possible to detach from traditional learning but it also turned into a constraint as the walls couldn't be used due to their mobility and technology rich surfaces.

#### Histories and practices intertwined in the work

The student teachers’ personal histories affected their work with children and at the university course. We could identify many supporting aspects regarding their previous knowledge and experience. The project-driven pedagogical approach was familiar for the students from similarly built previous courses. The course approach was also planned in the way that continuous negotiation for meanings was possible and embedded as a necessary activity in the pedagogical design of the course. The student teachers were familiar with the sociocultural perspective to language learning, positioning schoolchildren as active agents, aligning with PD practice. In a future workshop organized in the spirit of the PD tradition (Kensing and Madsen, 1991), the student teachers were eagerly sharing ideas and knowledge about existing, available, and future technologies, making their own firm background as users of social media and diverse technologies for learning and work prevalent. Despite their expertise in this area, they had, however, challenges in turning it usable in the school environment. The progress in student teachers’ understanding of their professional future as utilizing digital technology was marginal, which was visible in their talk and reflective writings.

There were also hindering factors regarding the histories. The student teachers studied children’s world and their development and (foreign) language resources through theoretical materials as well by making observations and interviews with children of the same age as the schoolchildren to be engaged in the emerging project. Despite the increased understanding of children’s technology-mediated language practices the student teachers still leaned towards traditional methods of language teaching due to their own previous experiences of their own language learning and in some cases also of the accustomed practices of teaching in the field.

Lack of previous knowledge of PD approach appeared to make it difficult for the student teachers to adopt a PD stance, which is not a surprise as such. They received support from the researchers in refining their original ideas for language learning activities on discussion lists and in class workshops so that the schoolchildren got an opportunity of being active agents in their learning with technologies. In their design work, the student teachers, however, rather focused on creating activating language learning activities than creating activities that would turn the schoolchildren’ stance to participatory designers as well, with the schoolchildren taking a look at the future and considering how their language learning practices with technologies might be different. Schoolchildren’ ideas were not drawn either on in the later phases of the process as the PD practice would have guided; they were not used by the student teachers for refining their concepts at the end of the course.

The fact that this was a university course shaped the process also in many ways.

Moving between the school and the university was impossible for many reasons - scheduling issues - time management - all the students also have their own timetables which are not necessarily compatible with the school's - in their major subject there was the absolutely obligatory attendance requirement in those days, and the lack of public transport, one cannot assume that they travel here and there at their own expense. (University teacher’s reflections)

As the schedule of the university course was tight, it was not possible to embed in the course flow opportunities for the student teachers to make contact with the schoolchildren before they were actually starting the project or continuously thorough the course. When the PD process was presented to the student teachers, they were expected to continue collaboration with the schoolchildren during their course. As this was not part of the main procedure of the course, most of them did not draw on this possibility. There were also practical difficulties for the students to go to the school, as it was not close to the university premises. Overall, there was a gap between the course and the school that resulted in schoolchildren’s ideas not being drawn on in the design process. Negotiation concerning children’s views was not embedded in the course structure well enough.

Technology researchers’ role in the course was unclear: they were not teachers of the course and had many other tasks and duties to take care of, preventing them from being fully engaged in guiding this work. This resulted in lack of support for the student teachers in their early engagement with PD. Even if the technology researchers educated them, the technology researchers could have taken more active role in pushing PD ideals in the course, discussing the reasoning, and suggesting alternative ways to do it. They could have also been more involved in the activities at the school during the theme week.

The school in question, particularly the schoolteachers, could also have been more involved in planning the entire project, but the university researchers and teachers ended up in doing the planning due to a variety of scheduling issues involved. The university teacher’s reflection sheds light on the situation on the course in relation to the university on the whole in the following quote:

Considering these [..] complexities even a PD professional wouldn't arrive at a different result because change would require a perspective switch in the curriculum - problem/project-based curriculum etc. and lots of time for breaking the tradition of language learning in the student teachers' minds - it won't happen during one course or even during their degree studies. (University teacher’s reflections)

University courses also have a set amount of credit units with which the students’ workload has to be aligned. Due to this, many PD activities were not implemented, or they were implemented only partially. A related factor is that in the course too little time was assigned for sense making between stakeholders for course teachers and student teachers to fully understand the circumstances at the school in the planning phase.

School setting was also shaping the work. The school was foregrounded in the university course as having its own practices, not necessarily relevant from the perspective of the digitalization and its impact on practices of learning and work more broadly. Nevertheless, the student teachers were explicitly guided in the university course to detach themselves from the print-oriented (often textbook-driven) practices of language learning at school and focus on language use and learning through meaningful activities from the schoolchildren’ perspective. The extract below from the university teacher’s lesson plans shows her efforts to help the student teachers to ideate language learning and teaching scenarios without too much adherence to the familiar environment of the classroom.

- Phase 1. Future workshop – flaps on the wall

1. Multimodal methods for moving from one place to another
2. Multimodal methods for interacting with each other
3. Places here and now, virtual etc.
4. Opportunities for foreign language contact
5. Possible technologies

- Phase 2. Multimodal pics (the above combined)

In the next meeting, the program will be as follows:

- presentations
- brainstorming on cases – activities with certain criteria (genuine participation, looking into the future, breaking old beliefs and myths, widening the scope of language learning, technology etc.)
- the participants will be divided into project teams responsible for the different research/design stations

(University teacher’s lesson plan)

Despite the guidance including the background work on schoolchildren’s perspectives in the orientation phase, there were different forces visible in the student teachers’ pedagogical designs, and the original designs often tended to follow the logic of traditional classroom exercises and tasks. The designs started to be geared towards activating the schoolchildren based on children’s own interests and preferences for learning when the student teachers were guided towards sociocultural views to learning, putting emphasis not only on linguistic precision but on authentic and creative language use as well.

The school premises and infrastructure affected the work in several ways, too, as discussed above in relation to the various agendas at play. The following example illustrates the delicate nature of schoolchildren’s interaction at school.

When the project involved hands-on work with the pupils, the student teachers saw that things don't always go as planned - activating pupils as designers did not take place - except for one group and also there minimally - because we saw how pupils all the time monitor each other's action and are shy (which is often vented as fooling around) when they don't know all the participants - pupils from higher grades, for example - so there's no genuine investment to design, even if that were included in the activities. (University teacher’s reflections)

The quote is related to one of the groups of student teachers arranging brainstorming with the schoolchildren during the school project, which was undermined by the open circumstances at the school not giving enough privacy for the children to reveal their thoughts. Furthermore, the mixing of pupils of different ages and grade levels may cause hindrances in genuine participation in ideation as various tensions between participants may arise, e.g., the familiar, safe community may dissolve. Young children need to be encouraged by specific methods and suitable spatial arrangements.

## Discussion

This paper contributes to the field of CSCW by introducing EPD as a novel methodological approach that integrates design with work practice learning and education. This study was motivated by our thought that practices common in PD could help future teachers in their work: enabling mutual reciprocal learning between student teachers as designers and schoolchildren as users while collaboratively experimenting and exploring within a design context. Yet, after realizing that while practicing in the real-life work context these students were also learning and experiencing in situ the digital transformation of learning and teaching work practices, the approach was reconsidered as EDP.

While EPD seems a promising methodological approach, its actual application needs transdisciplinary work to be able to integrate the educational fields and reach the transformation potential of the approach. Thus, our study also contributes to the CSCW discourse on transdisciplinary work. We respond to the call for revealing the complexities associated with transdisciplinary work (Khaled and Ingram, 2012). Instead of painting an overly positive picture, we show the messy and complex character of our endeavor to support the future development of the practice (Khaled and Ingram, 2012). We make it visible how the work is affected by the historical trajectories and practices in the fields involved as well as their complex entanglements reaching from situated action to societal dimensions.

### Transdisciplinary education with EPD for CSCW

As our first research question we asked what kind of resource educational participatory design provides for transdisciplinary education. We argue the notion of EPD holds great relevance for the CSCW community: EPD integrates design with work practice learning and education and therefore belongs to the core expertise of CSCW research and design. So far, transdisciplinary education has not been explicitly discussed in CSCW, while some interesting developments along these lines have emerged in architectural education (e.g., Iacucci and Wagner, 2003; Molin-Juustila et al., 2014). CSCW research has focused on real-life professional work contexts (Bjørn and Boulus-Rødje, 2015) while it also could have a significant impact by strengthening the digital technology skills and competencies of the future professionals (Wardrip et al., 2013). Work practice oriented CSCW combined with PD (see e.g., Kuutti, 2013; Karasti, 2001) should be considered valuable in educational contexts, preparing future professionals for the digital transformation of their work practice. Hence, CSCW researchers should take a stronger role in orchestrating transdisciplinary EPD processes for the needs of digital transformation of work, harnessing students, as future professionals, as practicing workers within real-life work context to initiate collaboration for designing the future work, enabled, and mediated with new digital technologies.

We identified a number of aspects that contributed to making it more challenging for the student teachers to adopt the PD stance (see also Kuure et al., 2016): the historical background and mindset of (language) teacher education, accustomed views on language teaching and learning, the role of digital technology in those, and the common panopticon classroom with the teacher as the hub for interaction and activities (Scollon and Scollon, 2004). Previous PD research has also pointed out how difficult it is for master’s level students to envision the transition from the traditional ways of working to new practices enhanced with new technologies without personally encountering the tensions between the old and nascent new (Molin-Juustila et al., 2014). From the perspective of EPD, these difficulties are thus not unexpected: as these student teachers had no prior familiarity with PD, it is understandable that adopting PD ideology and acting as professionals in PD after a brief training is a challenge (cf. Bødker and Iversen, 2002). In addition, we were expecting them to build design agency for digital transformation of their future teaching and learning work. Building professionalism in PD, or design agency, is, however, impossible to accomplish in a short time frame.

Nevertheless, we consider the PD spirit and principles gave a valuable basis for student teachers’ work with children, giving them a chance to question their accustomed ways of thinking. Noteworthy is that PD was only one part of the complex whole of organizing the course. Moreover, even though the course teachers had a good understanding of the PD approach and practices, they were themselves from the field of language studies and not professionals on PD nor on the design of digital technologies. Despite the good intentions, the intensive collaborative planning of the course, and the long history of transdisciplinary research within the research group, the complex entanglement of histories, practices, and varying agendas made it difficult to include children more vigorously in the process. The next section will further elaborate on these contextual factors.

### Transdisciplinary education with EPD intertwined with histories and practices

**Figure 2:**
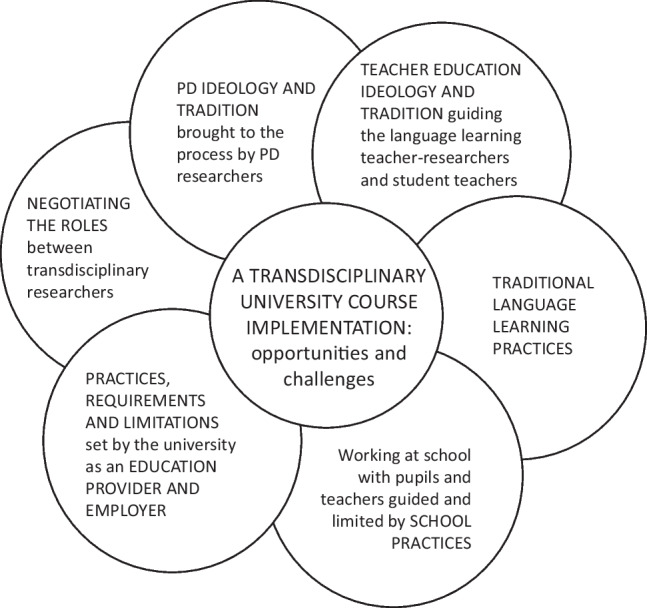
A transdisciplinary university course implementation in the crossroads of histories and practices.

Our second research question asks what kind of contextual factors are shaping the use of educational participatory design as a resource in transdisciplinary education. The significance of contextual factors affecting PD work has been understood for long (Kraft and Bansler, 1994), and in hindsight, it seems obvious that when aiming towards transdisciplinary education, researchers bringing their own practices to other fields need to be aware of the complex entanglement of histories and practices of the participating fields. We maintain that there were several histories and practices determining the path that the course could take in terms of how the PD principles were applied, and what kind of role the technology researchers were taking to encourage student teachers to adopt a more active PD stance with the school children, linked with the agendas and motivations of the different parties (see e.g., Krüger et al., 2021). Those concern both universities and comprehensive schools as institutions with their practices and histories as well as all the involved individuals with their life histories, experiences, accustomed practices, and expertise (Fig. 2). One can identify various practices, requirements, and limitations set by the university as an education provider and employer that had an important role here: Scheduling a university course, collaborating with the school, travelling to the school, and the student teachers’ overall workload all affected what seemed plausible to do in the course. The student teachers had limited contact with the schoolchildren, who they should have worked with as’users’ in the PD spirit. This was due to practical arrangements: contact with the school and schoolchildren needed to be established before the university course started, and the university course had its own schedule, tied to university teaching periods and teaching planning, and the work of the university teachers, whereas the school practices limited the work too: the school had its own schedule – there were only certain limited times and occasions when the students teachers had ‘access’ to the schoolchildren. As to the technology researchers’ role, they were university employees and had their own work (research and teaching) to take care of; it was not possible or expected for them to participate more actively in teaching this course; it was the work of the researchers on language studies, and they applied PD as they saw fit for the course.

From the viewpoint of this paper, it is more interesting, however, that we were able to achieve a new understanding of the possibilities and struggles of this transdisciplinary approach in education, showing the significance of diverse histories and practices shaping our work. CSCW researchers should take the initiative here, as interdisciplinary professionals in digital transformation of socio-technical work practices together with expertise on supporting collaboration, collaborative design, and participatory technology design. Yet, we need to remember that entering other fields is not straightforward. A deep understanding of their challenges, existing work practices and histories as well as mutual dialogue are needed to find out how CSCW disciplinary approaches, such as the PD stance, can be beneficial for them. Long-term transdisciplinary collaboration between fields is one way to acquire this, which will be discussed next.

### CSCW orchestrating transdisciplinary research, design, and education

We contribute to the CSCW discourse on transdisciplinary work. Multi-stakeholder and multi-discipline collaboration is at the heart of CSCW, and the field has been influenced and transformed by a range of disciplines through its lifespan (Schmidt and Bannon, 2013). However, there has been little discussion so far on what transdisciplinary design, or education entail in CSCW. Next some insights are offered based on our long-term transdisciplinary collaboration.

From the methodology perspective, in transdisciplinary research disciplinary boundaries need to be dissolved to construct methodologies tailored for solving the problem, with an emphasis on the integration of disciplinary epistemologies (Gibbons et al., 1994; Horlick-Jones and Sime, 2004). The methodologies also typically evolve in time (Wickson et al., 2006). In our case, we had already had long-term transdisciplinary research collaboration regarding the study of technology use in everyday life. A more explicit use of EPD provided an excellent way to combine our transdisciplinary interest in technology enhanced future. The research group jointly planned how the EPD approach could be used to renew the course, monitored the situation during the implementation of the course, and reflected on it afterwards. In a sense, integration of disciplinary epistemologies between the researchers/teachers was easy, as no conflicts emerged due to the joint history and shared research interests. The shared knowledge base included concept design, probes, and future workshops among others. These were familiar to the representatives of both disciplines, whereas the PD orientation was a novel addition that brought in the design orientation as well as the participatory aspect with learners as significant design participants.

However, the extended and repeated discussions about the outcomes of the project in the research group after the university course had ended, made it visible that there were still some differences in how the representatives from different disciplines understood the situation. While the technology researchers saw problems in the PD process (e.g., minimal engagement of users in the design process), the language researchers rather saw the university as a learning environment causing trouble (e.g., difficulty in moving from the learner position to the designer position, challenges in commitment, sticking to the traditional view of learning). We see this at least partly as an issue related to the participative nature of research collaboration stressed in transdisciplinary research; not only among researchers but involving also people somehow affected by the research, e.g., in problem formulation or in defining criteria, objectives, and resources needed for the endeavor and resolving the problem (Klein, 2004). Despite our long-term research collaboration within the research group, there clearly was a need for even clearer definition of aims, roles, and responsibilities between the researchers/teachers. Among the researchers, there was overlap in the knowledge base that has already been reported as easement for collaboration (cf. Godemann, 2008). However, the already acknowledged problems characterizing multi-, inter- and transdisciplinary research and practice, such as mismatches in goals, assumptions, practices, and understandings (cf. Adamczyk and Twidale, 2007; Murer et al., 2014; Rau et al., 2013) pictured also in our work, even if those did not turn out to be critical issues hindering our project.

We engaged in the collaboration and problem setting not only with children and their teachers but also the whole school as well as student teachers and researchers (cf. Klein, 2004; Wickson et al., 2006). In our work, knowledge domains from different fields were relatively easily merged (cf. Horlick-Jones and Sime, 2004; Klein, 2004; Lawrence, 2006; McClam and Flores-Scott, 2012). However, this could be seen to happen rather among the researchers than among the other participants. PD aims for mutual learning, democratic practices, and genuine participation of all affected (Luck, 2018; Greenbaum and Loi, 2012) were hindered by practicalities. We argue that to overcome the practical day-to-day issues a more participative culture in planning the teaching/learning in higher education would be needed in general, to make it a natural part of the process. We see that PD principles and practices would serve this well, and we argue that CSCW researchers should actively consider how they could change their own learning institutes’ teaching towards more participative nature. Even if PD has been seen as pivotal in systems design for decades and is included in the curricula of computing education of various kinds (most notably in HCI), its potential in the education of other disciplines remains weakly explored. In the digital age, transformation of work as well as education is required within several disciplines, including our own. We suggest increasing the visibility of EPD as an approach for the digital transformation of education. Language teacher education serves as an example here, while we maintain that it is probably difficult to find a discipline within which future professionals will not need skills and competencies to reflect on and engage in the digital transformations of their profession. Our approach offers an initial example along this path.

## Conclusion

This study and the EPD approach respond to the recent interest and call for transdisciplinary design and design education (Blevis et al., 2015) that have so far been hardly acknowledged in CSCW, even though definitely relevant. In this study, we offer a practical example of such an achievement – in a novel context, relevant in the future. There has been a blind spot so far in our understanding of PD potential in engaging in transforming the education of other disciplines and professions. Digital technology has entered all spheres of life and hence different professionals need to be equipped to appropriate, design, and modify digital technologies to suit their needs and purposes.

We suggest that PD should be seen as relevant for ‘designers’ representing various disciplinary and professional backgrounds. They should be able to adopt a designer stance to digital technology in the sense of considering various possibilities and consequences of digitalization in their work practice. Preferably, they should be able to adopt a participatory designer stance that would guide them to considering digital technology solutions not only for their own benefit but also for the benefit of their customers and users, or more broadly, their target group. CSCW and PD research and practice offer a multitude of means and tools for this type of work, but we should raise awareness of this recent trend and try to provide even better support for this work. It is important to try out what kind of practices work best with the ‘designers’ of other fields and consider how the existing tools could be improved to support these designers, or how new ones could be created.

We also believe that interdisciplinary CSCW researchers could be of value in the rapidly digitalizing world. They have expertise in how digital technologies transform work practices in general and how to approach this transformation methodologically. We consider this kind of expertise is needed in addition to the expertise of PD and HCI. We see EPD as a methodological approach situated in the middle of education and digital transformation of work. We propose that a CSCW point of view could play a significant role in refocusing transdisciplinary issues out from academic discussion, that revolves around the context of research and education, to real-life professional work contexts typically connected with in-situ (ethnographic) field studies, multiple sites of design, and interventionist agenda (Bjørn and Boulus-Rødje, 2015). CSCW research could have a significant impact by strengthening the digital technology skills and competencies of the future professionals of different kinds, even before they enter the workforce (Wardrip et al., 2013). We suggest that through EPD, CSCW researchers can contribute to this change in an essential way.

Regarding the limitations of the study, we did not originally plan the course to encompass all the important aspects of transdisciplinary work. We therefore recommend that those aiming at transdisciplinarity carefully consider the knowledge integration process (Godemann, 2008) and its aspects including (affecting) the attitudes of the participants, providing place for personal growth through relationships, and taking care of the needed resources (Wall and Shankar, 2008). Even if CSCW researchers have long ago acknowledged the prominence of multi-, inter-, and cross-disciplinary research and design practices, their integration into education has not been addressed in depth. Particularly transdisciplinary work practices – in design and education – require further studies. Hence, we welcome studies with more systematic use of the transdisciplinary approach, especially in taking initiative in promoting dialogue between CSCW and other fields concerning the use of the PD approach. In our case, the historical background and mindset of teacher education probably made it more challenging for the student teachers to adopt the participatory designer stance and work with schoolchildren as ‘users.’ Historical baggage also shapes and challenges the work among other professionals. Therefore, we warmly welcome researchers to take this challenge into account well in advance in their design projects.

We also suggest that future studies related to digital technology in a variety of contexts might benefit from the transdisciplinary perspective with a PD orientation examined in this study (e.g., in public-sector organizations, banks). Hence, we consider our findings useful for educational programs of different kinds, not only for language teacher education. In language teacher education, the aim is to educate future teachers not only to be competent in using digital technology themselves but also in prospecting the potential future transformations of their profession. This is a good example of a complex challenge for any educational program encountering the transition dynamics of their profession. The difficulties in learning how to take a EPD stance applies in the education of other professionals, too: one course on EPD is not enough in equipping them with a participatory designer stance, even if useful insights can be offered about that mindset. Longer-term educational efforts are needed in this respect. We think a problem-based curriculum that is used in some universities (e.g., Aalborg University in Denmark) may help to overcome many of the obstacles encountered in this study at the cross-roads of histories and practices, as the studies are organized on the basis of teamwork and projects on a long-term basis (Kolmos, 2010).

We also underscore that if participants do not have any background in PD tradition or design, more thorough training needs to be offered compared to our case, focusing particularly on the mindsets of design agency and a participatory designer. Relatively lightweight training of users may suffice for enabling participation in PD sessions, but for the designers who are to invite users as participants in design, more comprehensive education is needed, not only about the practicalities but the overall philosophy guiding the work. It can be motivated by the users’ (in our case, learners’) right to take part in decision-making affecting their life, while it also needs to be emphasized that the users’ valuable expertise and skills are needed for creating useful, usable as well as inspiring tools for them. However, the users need to be supported to be able to meaningfully contribute to the digital transformation of their lives, i.e., to the design process. A related challenge concerns reflection and learning that is to take place after work with users. Here, the ‘designers’ need support and guidance to be able to fully appreciate users’ input into visioning for future work and technology. The designer needs to acknowledge that the users’ expertise is central to success of the design process. However, it is also important to remember that the users’ experiences and understandings are guided by their own histories related to the field in question, in our case language education. These histories may be based on traditional and narrow practices of learning language matter rather than modern understandings of learning as meaningful interaction in a sociocultural environment. Enabling both users’ and designers’ thinking outside of the box is a future challenge to address.

To conclude, we see a lot of value in this study although the application of the PD approach was not fully accomplished: we managed to familiarize the student teachers with PD and give some practical experience on it. In their future professional life, this may prove out to be very beneficial in the sense of using digital technology in learning and teaching activities and in these teachers’ approach to their students – potentially more participatory practices may be applied in the classroom. This study offers an example on how one can introduce a PD and design orientation to experts representing other professions and disciplines. Future work should be able to avoid the most obvious shortcomings of our approach and inform the research community on the best practices in this kind of transdisciplinary design education.

## Footnotes

1 Multi-, inter-, and transdisciplinary work are used to refer to a continuum that includes increasing amounts of interaction and cross-fertilization among disciplines. Multidisciplinary work involves several disciplines working independently (either in parallel or sequentially) in an additive, unintegrated manner; interdisciplinary work entails reciprocal actions among disciplines, the solving of shared problems, and the integration of knowledge; and transdisciplinary work involves work across or beyond disciplines, transcending disciplinary boundaries and transforming disciplines (Choi and Pak 2006).
